# Supplementary material for: Virtual and Augmented Reality for Chronic Musculoskeletal Rehabilitation: A Systematic Review and Exploratory Meta-Analysis
Source: Bioengineering (Basel). 2025 Jul 8;12(7):745. doi: 10.3390/bioengineering12070745 (PMC12292062; doi:10.3390/bioengineering12070745)
Supplement: Supplementary file 1 [file bioengineering-12-00745-s001.zip › bioengineering-3726201-supplementary.pdf]

Supplementary Table S1. Full Search Syntax per Database

Database	Search Period	Search String (Boolean Syntax)	Limits / Filters Applied
**PubMed**	Inception – 31 Mar 2024	("virtual reality"[Title/Abstract] OR "VR"[Title/Abstract] OR "augmented reality"[Title/Abstract] OR "AR"[Title/Abstract] OR "extended reality"[Title/Abstract] OR "XR"[Title/Abstract] OR "exergaming"[Title/Abstract] OR "serious games"[Title/Abstract] OR "Kinect"[Title/Abstract] OR "Nintendo Wii"[Title/Abstract]) AND ("musculoskeletal"[Title/Abstract] OR "osteoarthritis"[Title/Abstract] OR "fibromyalgia"[Title/Abstract] OR "chronic pain"[Title/Abstract] OR "back pain"[Title/Abstract] OR "low back pain"[Title/Abstract] OR "shoulder dysfunction"[Title/Abstract] OR "patellofemoral pain"[Title/Abstract] OR "TKA"[Title/Abstract]) AND ("rehabilitation"[Title/Abstract] OR "physical therapy"[Title/Abstract] OR "physiotherapy"[Title/Abstract] OR "exercise therapy"[Title/Abstract])	Filters: Humans, English, Adults ≥18 years, RCTs
**Scopus**	Inception – 31 Mar 2024	TITLE-ABS-KEY ("virtual reality" OR "VR" OR "augmented reality" OR "AR" OR "extended reality" OR "XR" OR "exergaming" OR "serious games" OR "Kinect" OR "Nintendo Wii") AND TITLE-ABS-KEY ("musculoskeletal" OR "osteoarthritis" OR "fibromyalgia" OR "chronic pain" OR "back pain" OR "low back pain" OR "shoulder dysfunction" OR "patellofemoral pain" OR "TKA") AND TITLE-ABS-KEY ("rehabilitation" OR "physical therapy" OR "physiotherapy" OR "exercise therapy")	Document Type: Article; Language: English; Subject Area: Medicine, Health Professions
**PEDro**	Inception – 31 Mar 2024	Abstract/Title contains: (virtual reality OR augmented reality OR exergaming OR Kinect OR Wii) AND (musculoskeletal OR pain OR arthritis OR fibromyalgia OR rehabilitation)	Filters: Clinical trial, RCT; English; Human studies
**Cochrane CENTRAL**	Inception – 31 Mar 2024	("virtual reality" OR "VR" OR "augmented reality" OR "AR" OR "exergaming" OR "Kinect" OR "Nintendo Wii") in Title, Abstract, or Keywords AND ("musculoskeletal" OR "osteoarthritis" OR "fibromyalgia" OR "chronic pain" OR "rehabilitation" OR "physiotherapy")	Filters: Trials only; Adult participants; English
**Google Scholar / OpenGrey**	Jan 2010 – Mar 2024	("virtual reality" OR "augmented reality" OR "exergaming") AND ("chronic musculoskeletal" OR "rehabilitation")	No filters applied; hand-screened based on title/abstract relevance
**ClinicalTrials.gov**	Inception – 31 Mar 2024	Condition: musculoskeletal; Other terms: virtual reality OR augmented reality OR exergaming; Study type: Interventional; Recruitment: All	Filters: Adults; Study type: RCT; Status:

Database	Search Period	Search String (Boolean Syntax)	Limits / Filters Applied
			Completed or Ongoing

PubMed
("virtual reality"[tiab] OR "VR"[tiab] OR "augmented reality"[tiab] OR "AR"[tiab] OR "extended reality"[tiab] OR "XR"[tiab] OR "exergaming"[tiab] OR "serious games"[tiab] OR "Kinect"[tiab] OR "Nintendo Wii"[tiab])
AND
("musculoskeletal"[MeSH Terms] OR "osteoarthritis"[MeSH Terms] OR "fibromyalgia"[MeSH Terms] OR "chronic pain"[MeSH Terms] OR "low back pain"[MeSH Terms] OR "shoulder pain"[MeSH Terms] OR "patellofemoral pain"[tiab] OR "TKA"[tiab])
AND
("rehabilitation"[MeSH Terms] OR "physical therapy"[MeSH Terms] OR "exercise therapy"[MeSH Terms]) Scopus
TITLE-ABS-KEY("virtual reality" OR "VR" OR "augmented reality" OR "AR" OR "extended reality" OR "XR" OR "exergaming" OR "serious games" OR "Kinect" OR "Nintendo Wii")
AND
TITLE-ABS-KEY("musculoskeletal" OR "osteoarthritis" OR "fibromyalgia" OR "chronic pain" OR "low back pain" OR "shoulder dysfunction" OR "patellofemoral pain" OR "TKA")
AND
TITLE-ABS-KEY("rehabilitation" OR "physical therapy" OR "physiotherapy" OR "exercise therapy")
PEDro
(virtual reality OR VR OR augmented reality OR AR OR exergaming OR Kinect OR Wii)
AND
CENTRAL (Cochrane Library)
("virtual reality" OR "VR" OR "augmented reality" OR "AR" OR "extended reality" OR "XR" OR "exergaming" OR "Kinect" OR "Wii") in Title Abstract Keyword
AND
("musculoskeletal" OR "osteoarthritis" OR "fibromyalgia" OR "chronic pain" OR "low back pain" OR "TKA") in Title Abstract Keyword
AND
("rehabilitation" OR "physical therapy" OR "exercise therapy") in Title Abstract Keyword
(musculoskeletal OR osteoarthritis OR fibromyalgia OR chronic pain OR back pain OR shoulder pain OR TKA)

Supplementary Table S3. GRADE Evidence Profile for the Outcome "Pain Reduction"

Outcome	Risk of Bias	Inconsistency	Indirectness	Imprecision	Publication Bias	Certainty of Evidence
Pain Reduction (VAS)	Low to Moderate	None ($I^2 = 0\%$)	None	Moderate	Suspected	**Moderate**

Explanatory Notes:

- **Risk of Bias**: The majority of included trials were rated as good quality based on PEDro (mean 6,31/10) and Downs & Black (mean 22,8/28) assessments. Lack of participant blinding was common but considered unlikely to significantly impact outcome estimates.
- **Inconsistency**: The pooled analysis yielded no statistical heterogeneity ($I^2 = 0\%$), suggesting consistent treatment effects across trials.

- **Indirectness**: All studies targeted adult patients with chronic musculoskeletal disorders and employed XR-based interventions in line with the review objective. No serious indirectness identified.
- **Imprecision**: The relatively small number of included RCTs (n = 8) and wide confidence intervals in some cases lower the precision of pooled estimates.
- **Publication Bias**: Not formally assessed due to limited study number (n < 10); however, the prevalence of positive findings suggests a possibility of reporting bias.
- **Overall Certainty**: The evidence was rated as **moderate** due to minor limitations in risk of bias, imprecision, and potential publication bias.

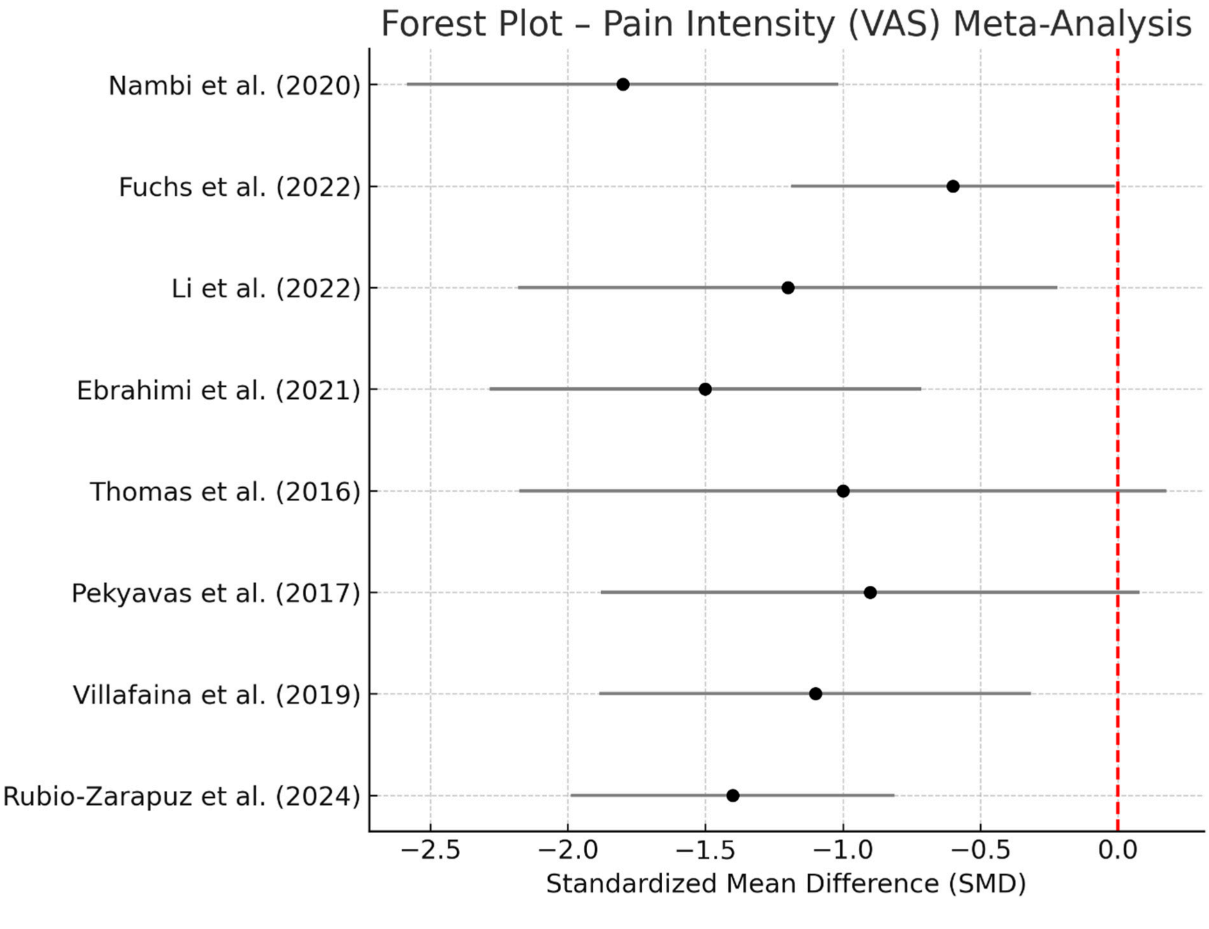

Supplementary Table S2 Summary of Studies Included in the Exploratory Meta-Analysis on Pain Outcomes (VAS)

Study	SMD	Standard Error	Technology	Population	95% CI (Lower)	95% CI (Upper)	Weight (%)
Nambi et al. (2020)	–1.80	0.40	VR immersive	Post-traumatic OA	–2.58	–1.02	14.84
Fuchs et al. (2022)	–0.60	0.30	VR immersive	Total Knee Arthroplasty	–1.19	–0.01	17.60

Study	SMD	Standard Error	Technology	Population	95% CI (Lower)	95% CI (Upper)	Weight (%)
Li et al. (2022)	–1.20	0.50	AR immersive	Post-op Knee Rehabilitation	–2.18	–0.22	11.34
Ebrahimi et al. (2021)	–1.50	0.40	Exergaming	Patellofemoral Pain	–2.28	–0.72	14.84
Thomas et al. (2016)	–1.00	0.60	VR immersive	Chronic Low Back Pain	–2.18	0.18	9.61
Pekyavas et al. (2017)	–0.90	0.50	Exergaming	Shoulder Dysfunction	–1.88	0.08	11.34
Villafaina et al. (2019)	–1.10	0.40	Exergaming	Fibromyalgia	–1.88	–0.32	14.84
Rubio-Zarapuz et al. (2024)	–1.40	0.30	VR + Neuromodulation	Fibromyalgia	–1.99	–0.81	15.60
